# Dietary supplementation with canthaxanthin and 25-hydroxycholecalciferol on the incubation performance and fertility of European quail breeders

**DOI:** 10.1016/j.psj.2022.101823

**Published:** 2022-03-08

**Authors:** L.P. Bonagurio, A.E. Murakami, F.K. Cruz, I.N. Kaneko, E. Gasparino, C.A.L. Oliveira, C.A. Lozano-Poveda, C.C. Silva, T.C. Santos

**Affiliations:** ⁎ Department of Animal Science, State University of Maringá, Av. Colombo, 5790, Maringá, Paraná 87020-900, Brazil; † DSM Nutritional Products, São Paulo, Brazil

**Keywords:** carotenoids, hatching, pasgar score, sperm, vitamin D_3_

## Abstract

This study assessed the effects of combined supplementation with canthaxanthin (**Cx**) and 25-hydroxycholecalciferol (**25-OH-D_3_**) on incubation performance, fertility, and chick quality in European quail breeders. A total of 240 birds were distributed in a completely randomized design with 5 diets and 8 replicates. The animals were fed a basal diet containing 50 µg of vitamin D_3_ or the basal diet supplemented with 3 ppm Cx and 34.5 µg 25-OH-D_3_, 6 ppm Cx, and 69 µg 25-OH-D_3_, 9 ppm Cx and 103.5 µg 25-OH-D_3_, or 12 ppm Cx and 138 µg 25-OH-D_3_. Incubation performance was analyzed in 2 periods (32 and 38 wk). Breeders aged 32 wk produced eggs with higher hatchability (*P* = 0.024), hatchability of fertile eggs (*P* = 0.026) and lower initial plus mid embryonic mortality (*P* = 0.021), whereas 38-week-old breeders generated chicks with a higher length at hatching (*P* < 0.001) and lower final plus pipped embryonic mortality (*P* = 0.021). In both age groups, Cx + 25-OH-D_3_ levels had a quadratic effect on egg fertility (*P* < 0.001), hatchability of total (*P* < 0.001), and fertile eggs (*P* < 0.001). The fertility and the number of sperm cells in the perivitelline membrane was analyzed in two periods (26 and 40 wk). A quadratic effect of diet and days after mating on both parameters (*P* < 0.05) was observed. Eggs from supplementing breeders showed a high fertility (*P* < 0.001) and sperm cell counts (*P* < 0.001) for up to 7 and 3 d after mating, respectively, then the control group. Moreover, the supplementation of quail breeder diets with 6 ppm Cx + 69 µg 25-OH-D_3_ enhances sperm cell longevity in sperm storage tubules, hatchability of total and fertile eggs, fertility, and chick quality, especially in older quail's breeders and reduces embryonic mortality.

## INTRODUCTION

Canthaxanthin (**Cx**) combined with 25-hydroxycholecalciferol (**25-OH-D_3_**) is a commercial additive commom applied in diets of poultry. Supplementation of breeder diets with synthetic carotenoids, such as Cx, successfully increase the concentration of antioxidant compounds and, consequently, reduces the formation of reactive oxygen species (Surai et al., 2016), in the oviduct trait (Bonagurio et al., 2020), plasma and serum (Karadas et al., 2016), and cell membranes (Mathimaran et al., 2021). Although, the Cx supplementation inhibits polyunsaturated fatty acids (**PUFAs**) peroxidation in sperm cell membranes, increasing sperm cell survival and flock fertility (Rosa et al., 2012).

Female birds can store sperm cells in their reproductive tract following copulation. Sperm cells are sequestered, selected, and stored for up to 2 wk in specialized structures called sperm storage tubules, located at the uterovaginal junction of the oviduct epithelium (Bakst, 2011; Santiago-Moreno and Blesbois, 2020). This process allows for sustained female fertility, as spermatozoa are slowly but constantly released to fertilize the eggs (Bakst, 1981).

The survival of spermatozoa during storage depends greatly on the prevention of PUFAs peroxidation (When et al., 2020) by the synergistic action of antioxidant nutrients and antioxidant defense enzymes (Surai, 2012). The structural, function integrity, and viability of stored sperm cells decreases with time, mainly because of oxidative damage (Bisht et al., 2017). Reactive oxygen species lead to the peroxidation of PUFAs in the sperm cell membrane, causing membrane disorganization and cell death (Alahmar, 2019).

Part of the Cx absorbed by the female bird is deposited in the yolk, from where it is transported to embryonic tissues (Schneider, 2016). Thus, Cx supplementation can decrease oxidative stress during embryonic development, improving hatchability, embryo survival, and offspring viability (Rosa et al., 2012; Araujo et al., 2019).

Supplementation of breeders with 25-OH-D_3_ also has beneficial effects on the reproductive system. This vitamin D metabolite has a high absorption rate and increases the efficiency of calcium absorption in the intestine. As a consequence of an increased circulating calcium levels, breeders produce eggs with a thicker shell (Bonagurio et al., 2020), minimizing the risk of breakage and contamination of the egg with infectious agents (Bar et al., 1980).

Like Cx, the 25-OH-D_3_ is transported to the yolk and embryo during development with higher efficiency than the vitamin D_3_ (Coto et al., 2010). These metabolite increases calcium concentrations in embryonic tissues and stimulates bone metabolism and immunological parameters (Vazquez et al., 2018), which in turn reduces embryonic mortality and improves hatchability and chick quality (Saunders-Blades and Korver, 2015) Landy and Toghyani (2018). reported that inadequate Ca-P supplementation with cholecalciferol significantly decreased the incidence of avian tibial dyschondroplasia, and tibia ash compared with broiler fed with the diet contain only 1α-Hydroxyvitamin D_3_ (1α(OH)D_3_).

The present study aimed to examine the effects of combined supplementation with Cx and 25-OH-D_3_ on incubation performance, chick quality, fertility, and spermatozoa quantity analyzed in different ages of European quail breeders.

## MATERIAL AND METHODS

The experiment was approved by the Animal Ethics Committee of the State University of Maringá, Paraná, Brazil (protocol no. 784611115).

### Animals, Housing, and Experimental Design

Meat-type quail breeders aged 24 wk were selected on the basis of their body weight (females = 292.01 ± 17.82 g, males = 251.84 ± 19.07 g) and egg production (90 ± 5%). Quail were housed in 25 × 39 cm laying cages made of galvanized wire with ad libitum access to water and feed under a 17L:7D photoperiod. The experiment was conducted during the spring, when birds were aged 26 to 42 wk. The animals were given a 14-d adaptation period prior to the initiation of the experiment.

A total of 240 quail were distributed into a completely randomized design consisting of 5 treatments and eight replications. Each cage of 6 birds (4 females and 2 males) was considered an experimental unit. Birds in the same cage were fed the same diet. Treatments were as follows: a basal diet containing 50 µg of vitamin D_3_ or the same diet supplemented with 3 ppm Cx and 34.5 µg 25-OH-D_3_, 6 ppm Cx and 69 µg 25-OH-D_3_, 9 ppm Cx and 103.5 µg 25-OH-D_3_, or 12 ppm Cx and 138 µg 25-OH-D_3_ (Table 1). All experimental diets were isoenergetic and isoproteic, based on corn and soybean meal, and formulated according to Rostagno et al. (2011). A vitamin-mineral premix and a commercial supplement containing Canthaxanthin (Cx) and 25-hydroxycholecalciferol (25-OH-D_3_) (MaxiChick, DSM, Nutritional Products, Brazil) provided the recommended levels of vitamin D_3_.

**Table 1:** Ingredients and chemical composition of the experimental diets.

Ingredients (%)	Diets^1^				
	Basal	3Cx	6Cx	9Cx	12Cx
Corn	51.76	51.76	51.76	51.76	51.76
Soybean meal (45% protein)	37.03	37.03	37.03	37.03	37.03
Limestone	5.85	5.85	5.85	5.85	5.85
Soybean oil	2.88	2.88	2.88	2.88	2.88
Dicalcium phosphate	1.29	1.29	1.29	1.29	1.29
Vitamin–mineral premix^2^	0.40	0.40	0.40	0.40	0.40
Common salt	0.33	0.33	0.33	0.33	0.33
dl-Methionine (98.5%)	0.20	0.20	0.20	0.20	0.20
l-Lysine (76.5%)	0.06	0.06	0.06	0.06	0.06
Inert (kaolin)	0.20	0.15	0.10	0.05	0.00
MaxiChick^3^	0.00	0.05	0.10	0.15	0.20

Estimated composition					

Crude protein (%)	21.00	21.00	21.00	21.00	21.00
Metabolizable energy (kcal/kg)	2.850	2.850	2.850	2.850	2.850
Calcium (%)	2.700	2.700	2.700	2.700	2.700
Chlorine (%)	0.248	0.2 48	0.248	0.248	0.248
Available phosphorus (%)	0.350	0.350	0.350	0.350	0.350
Potassium (%)	0.828	0.828	0.828	0.828	0.828
Sodium (%)	0.150	0.150	0.150	0.150	0.150
SID^4^ lysine (%)	1.097	1.097	1.097	1.097	1.097
SID^4^ methionine (%)	0.480	0.480	0.480	0.480	0.480
SID^4^ methionine + cystine (%)	0.767	0.767	0.767	0.767	0.767
Canthaxanthin (mg/kg)^5^	2.21	3	6	9	12
25-Hydroxycholecalciferol (µg/kg)	-	34.5	69.0	103.5	138.0
Vitamin D_3_ (µg/kg)	50	50	50	50	50

1 Basal diet (containing 50 µg vitamin D_3_); 3Cx, basal diet supplemented with 3 ppm Cx and 34.5 µg 25-OH-D_3_; 6Cx, basal diet supplemented with 6 ppm Cx and 69.0 µg 25-OH-D_3_; 9Cx_,_ basal diet supplemented with 9 ppm Cx and 103.5 µg 25-OH-D_3_, and 12Cx, basal diet supplemented with 12 ppm Cx and 138.0 µg 25-OH-D_3_ per kilogram.

2 Provided per kilogram of premix: vitamin A, 2,500,000 IU; vitamin D_3_, 500,000 IU; vitamin E, 5,000 IU; vitamin B1, 625 mg; vitamin B2, 1,500 mg; vitamin B6, 1,250 mg; vitamin B12, 5,000 µg; vitamin K3, 750 mg; pantothenic acid, 3,000 mg; niacin, 6,000 mg; folic acid, 250 mg; biotin, 50.0 mg; choline, 75 g; butylated hydroxytoluene, 1,000 mg; zinc, 13 g; iron, 13 g; manganese, 15 g; copper, 3,000 mg; cobalt, 50 mg; iodine, 250 mg; selenium, 63 mg.

3 MaxiChick (DSM, São Paulo, Brazil) is a dietary supplement composed of Cx (6 mg/kg) and 25-OH-D_3_ (69 µg/kg).

4 SID, standardized ileal digestible.

5 Cx contents were analyzed in the basal diet and estimated for the other diets.

### Quantification of Cx

Cx contents in the basal diet were determined by high-performance liquid chromatography (CBO Laboratories, São Paulo, Brazil). Values of Cx in the other diets were estimated using linear and quadratic regression from the results.

### Incubation Performance

Eggs from birds (n = 40 per experimental treatment) aged 32 and 38 wk were analyzed for incubation performance. All eggs produced by each experimental unit were collected for five days and stored in a refrigerated room (20°C). The eggs were incubated in an automatic incubator at 37.4°C and 60% relative humidity for 348 h, transferred to the hatcher, and incubated at 37.0°C and 70% relative humidity for a further 56 h. After incubation, all unhatched eggs were opened to determine egg fertility, infertility, and embryonic mortality. Embryonic mortality was categorized into initial (1–5 d) plus mid (6–11 d) (M1) and late (12–17 d) plus pipped eggs (M2).

### Chick Quality

The quality of newly hatched chicks (n = 40 per experimental diet) was analyzed by the Pasgar score method adapted from Boerjan (2006), a 10-point scale that measures reflex, navel closure, legs, beak, and belly characteristics. This subjective assessment was performed by a single person to avoid interexaminer variation. Body weight was determined using a digital scale. Chick length was determined by measuring the distance from the beak to the middle toe with a measuring tape.

### Analysis of Sperm–Egg Interaction

Quail couples (n = 9 couple per treatment) used in this experiment were fed the experimental diets since the beginning of the study period. Females were kept isolated from males for at least 15 d so as not to have live spermatozoa in their oviduct. When the birds were aged 26 and 40 wk, a male was placed in the female's cage for 24 h. Eggs were collected daily for 12 d and stored at 4°C.

Egg fertility was estimated by the morphology of the germinal disc (Kosin, 1945). The number of spermatozoa in the perivitelline space was determined. Briefly, 1.5 cm^2^ pieces of the germinal disc region containing the perivitelline membrane were cut and washed in 1% NaCl solution to remove the yolk. Specimens were mounted on microscope slides, stained with 5 L of 5 µmol 4’,6-diamidino-2-phenylindole dihydrochloride (**DAPI**) (Wishart, 1997), covered with a coverslip, and sealed with nail polish. The number of sperm cells in 10 fields (7.50 mm^2^) was counted under a fluorescence microscope at 10 × magnification and expressed as spermatozoa/mm^2^.

### Statistical Analysis

All statistical analyses were performed at the 0.05 significance level using SAS version 9.0 (SAS Institute, Cary, NC). Chick weight, length, and Pasgar score were subjected to analysis of variance (**ANOVA**). Regression analysis was performed using the general linear model (**GLM**) procedure of SAS. Other data did not follow a normal distribution and were analyzed using the GENMOD procedure of SAS with a BINOMIAL, GAMMA or POISSON distribution, according to data behavior.

Hatchability of total and fertile eggs, total mortality, fertility and infertility of incubated eggs, and fertility based on germinal disc morphology were analyzed in the function of 5 diets and 2 periods (32 and 38 wk) using a binomial distribution with better adjustment to the LOGIT link function.

The embryo mortality data classified as early, medium, late and pipped followed a LOG link function and PREDICT statement was used to assess differences among categories by the least square's method (*P* > χ2).

The number of spermatozoa to an egg be fertile and the probability of fertility after one day of male and female mating were estimated as a function of time. These data had a GAMMA distribution and better adjustment of data with an INVERSE link function.

The total number of spermatozoa in the perivitelline membrane over an area of germinal disc were analyzed in function of 5 diets and 2 different periods (26 and 40 wk) by Poisson distribution with better adjustment of data to LOG function.

## RESULTS AND DISCUSSION

### Incubation Performance

Eggs from 32- and 38-wk-old quail breeders were analyzed to the incubation performance and results are showed in Table 2. There were no interaction effects (*P* > 0.05) between breeder age and Cx + 25-OH-D_3_ supplementation on incubation parameters (Table 2).

**Table 2:** Mean observed of incubation performance variables of European quail at 32- and 38-weeks eggs supplemented with Cx + 25-OH-D_3_.

Breeder age	Hatchability, %	Fertility, %	Infertility, %	Hatchability fertile eggs, %	Mortality, Fertile eggs, %	Mortality, %	
						M1^2^	M2^3^
32 wk	77.66^A^	92.76	7.24	83.58^A^	16.42^B^	27.50^B^	72.50^A^
38 wk	69.60^B^	90.66	9.34	76.22^B^	23.78^A^	45.83^A^	54.17^B^

Diets^1^							

Basal (50 µg D_3_)	66.05	85.61	14.39	76.44	23.56	54.17	45.83
3 Cx + 34.5 µg 25-OH-D_3_	83.71	97.89	2.11	85.61	14.39	16.67	83.33
6 Cx + 69 µg 25-OH-D_3_	83.25	96.47	3.53	86.32	13.68	25.00	75.00
9 Cx + 103.5 µg 25-OH-D_3_	72.42	92.40	7.60	78.49	21.51	44.44	55.56
12 Cx + 138 µg 25-OH-D_3_	62.70	86.19	13.81	72.64	27.36	43.06	56.94

Mean	73.63	91.71	8.29	79.90	20.10	36.67	63.33
SEM	1.931	1.137	1.137	1.558	1.558	4.629	4.629

Variation sources				P-value			

Age	0.024	0.798	0.798	0.026	0.026	0.014	0.014
Linear	<0.001	<0.001	<0.001	0.033	0.013	0.669	0.669
Quadratic	<0.001	<0.001	<0.001	0.009	0.009	0.734	0.734
Interaction	0.748	0.417	0.846	0.959	0.959	0.829	0.829

Variables	Value estimated^4^				Vertex (ẏ^5^)	Cx (ppm)	25-OH-D_3_ (µg)

Hatchability	β = 0.820 + 0.276Cx − 0.025Cx²				82.94	5.57	64.05
Fertility	β = 1.955 + 0.459Cx − 0.040Cx²				96.34	5.74	66.01
Infertility	β = - 1.955 − 0.459Cx + 0.040Cx²				3.66	5.74	66.01
Hatchability of fertile eggs	β = 1.335 + 0.178Cx − 0.017Cx²				85.83	5.24	60.26
Mortality of fertile eggs	β = - 1.335 − 0.178Cx + 0.017Cx²				14.17	5.24	60.26

1 Basal diet (BD), containing 50 µg vitamin D_3_; 3Cx, BD supplemented with 3 ppm Cx and 34.5 µg 25-OH-D_3_; 6Cx, BD supplemented with 6 ppm Cx and 69.0 µg 25-OH-D_3_; 9Cx, BD supplemented with 9 ppm Cx and 103.5 µg 25-OH-D_3_, and 12Cx, BD supplemented with 12 ppm Cx and 138.0 µg 25-OH-D_3_ per kilogram.

2 M1 = initial plus medium mortality (0–8 days).

3 M2 = final plus pipped mortality (9–17 days).

4 *ŷ = e^β^*/(1 + *e^β^*)

5 Maximum or minimum ŷ values.

A-B Capital letters represent the differences between the mean ages that were detected (*P* < 0.05) by the least squares mean test.

Breeder age had no effect on fertility and infertility. However, breeders with 32 wk old showed higher total hatchability (*P* < 0.05), hatchability of fertile eggs (*P* < 0.05) and final plus pipped mortality (*P* < 0.05) when compared with breeders with 38 wk. On the other hand, breeders with 38 wk old showed higher mortality of fertile eggs (*P* < 0.05) and initial plus mean mortality (*P* < 0.05) (Table 2).

Cx + 25-OH-D_3_ supplementation had a linear and quadratic increasing effect (Table 2) on hatchability (*P* < 0.01), fertility eggs (*P* < 0.01) and hatchability of fertile eggs (*P* < 0.01) (Table 2)

**Figure 1:**
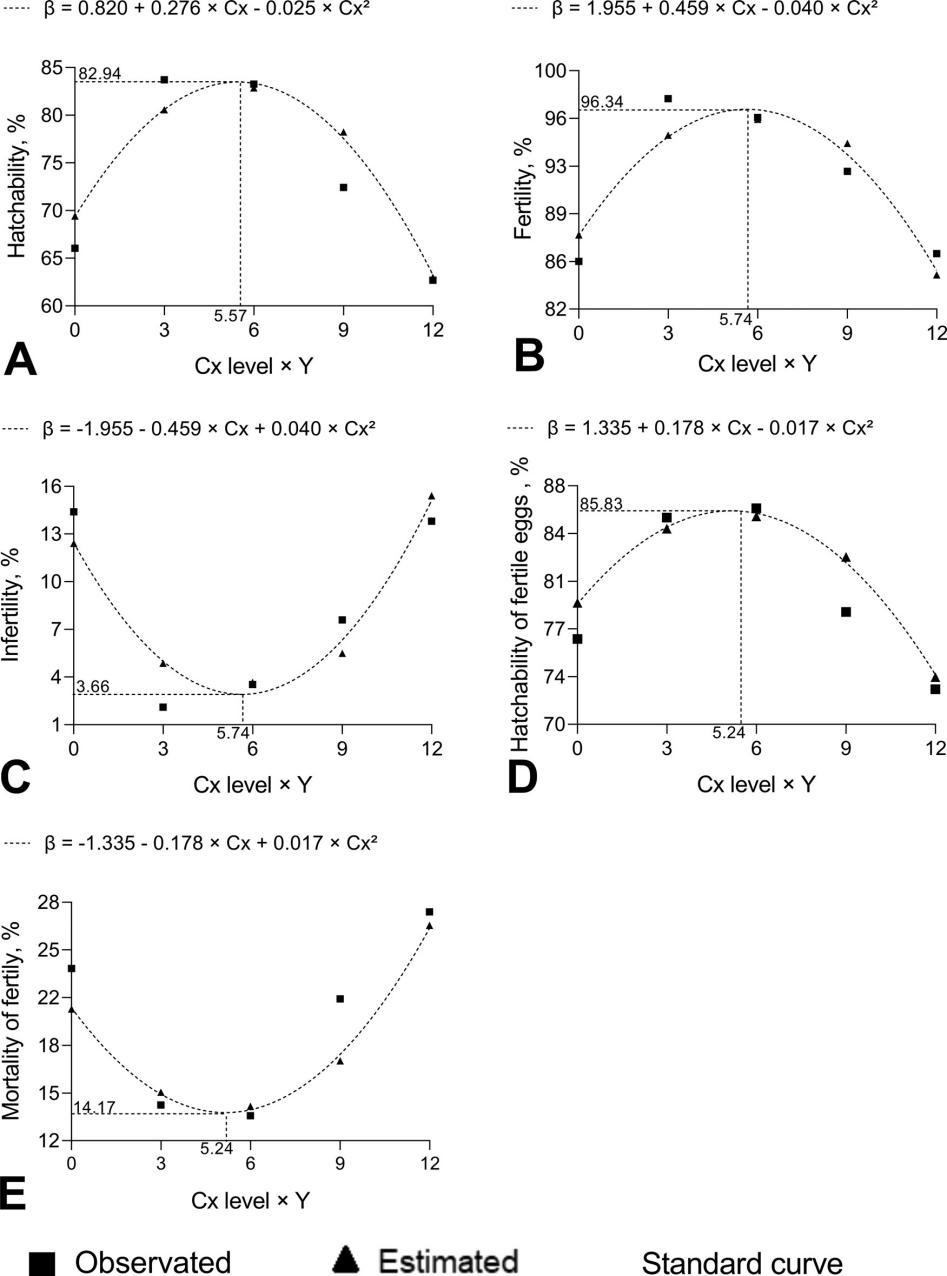
Graphics from incubation performance analysis. Diets: Basal diet (BD), containing 50 µg vitamin D3); 3Cx, BD supplemented with 3 ppm Cx and 34.5 µg 25-OH-D_3_; 6Cx, BD supplemented with 6 ppm Cx and 69.0 µg 25-OH-D_3_; 9Cx, BD supplemented with 9 ppm Cx and 103.5 µg 25-OH-D_3_, and 12Cx, BD supplemented with 12 ppm Cx and 138.0 µg 25-OH-D_3_ per kilogram.

The maximum hatchability (82.94%), fertility (96.34 %) and hatchability of fertile eggs (85.83 %), could be achieve with 5.57 ppm Cx + 64.05 µg 25-OH-D_3_, 5.74 ppm Cx + 66.01 µg 25-OH-D_3_ and 5.24 ppm Cx + 60.26 µg 25-OH-D_3_, respectively (Table 2 and Figure 1A, B, and D, respectively).

On the other hand, infertility (*P* < 0.01) and mortality of fertile eggs (*P* < 0.01) decreased quadratically to Cx + 25-OH-D_3_ levels until reaching 3.66 % and 14.17 % at 5.74 ppm Cx + 66.01 µg 25-OH-D_3_, and 5.24 ppm Cx + 60.26 µg 25-OH-D_3,_ respectively (Table 2 and Figure 1C, and E, respectively).

The chicks must spend large amounts of energy during hatching, which increases the oxidation of metabolic fuels. As chicks come into contact with an atmosphere rich in oxygen, the rate of oxidation reactions is further increased (Surai et al., 2006; Surai et al., 2016). Therefore, the maternal diet-mediated improvement in the bone, antioxidant system and immune development of embryos is an important factor contributing to hatching success.

The yolk is the embryo's sole source of nutrients during incubation. The amount of calcium transported to the yolk is highly associated with the levels of vitamin D_3_ and 25-OH-D_3_ (r = 0.99 and r = 0.94, respectively) in maternal diets (Mattila et al., 1999). Like calcium, the amount of Cx transported to the yolk is highly associated with the levels of Cx in maternal diets (Rosa et al., 2017).

The increase in hatchability of total and fertile eggs, and fertility of breeders, as well as the reduction in the embryonic mortality and infertile eggs, may have been due in part to the action of Cx and 25-OH-D_3_ to provide improvements in the bone structure and antioxidant system of breeders, which enhances chick's development.

In the metabolism, Cx eliminates free radicals, thereby decreasing the peroxidation of lipid nutrients, such as vitamin E (Surai et al., 2003; Surai, 2012; Surai et al., 2016). In addition, Cx enhances the defense of the antioxidant system against the Reactive oxygen species (**ROS**) that oxidized the PUFAs of spermatozoa localized in the oviduct trait and duct seminiferous (Bonagurio et al., 2020), as well as in the yolk egg. Therefore, dietary supplementation of Cx can enhance the antioxidant defense system against the peroxidation of PUFAs caused mainly by the ROS in several tissues of breeders, embryo, as well in the first live week of chick (Surai, 2002).

Mounting evidence has shown beneficial effects of supplementation of maternal diets with 6 ppm Cx on the antioxidant status of offspring. Cx in embryonic tissue acts scavenging free radicals and peroxides, and the recycling of vitamin C and E. Thus, Cx is able to alleviate oxidative stress in embryo metabolism during incubation and after hatching (Araujo et al., 2019).

Maternal diet added with different levels of Cx, promote a reduction in lipid peroxidation of the liver of chicks aged 1 and 7 d (Surai et al., 2003) Robert et al. (2008). also showed a reduction in malondialdehyde concentration in the liver of 1-day-old broiler chicks. Besides, the added 6 ppm Cx to the diets of broiler breeders (47 wks), promote a decrease in free radicals in blood serum and egg yolk (Zhang et al., 2011).

Whereas 25-OH-D_3_ could improve the calcium homeostasis in the breeder's metabolism, because is efficiently absorbed and increases calcium concentrations in the eggshell, improving shell resistance and density, promoting a reduction in the infections caused by pathogens Coto et al. (2010). showed that the addition of 25-OH-D_3_ in breeder hens’ diet improved egg-shell thickness, hen day production and egg mass. Therefore, 25-OH-D_3_ acts to stimulate embryonic bone development and reduce embryo mortality (Fatemi et al., 2020).

Our previously published study has evidenced that dietary breeder supplemented with 7.64 ppm of Cx + 87.88 µg of 25-OH-D_3_ showed a lower calcium plasma concentration (10.46 mg/dL), whereas added of 5.5 ppm of Cx + 60.03 µg of 25-OH-D_3_ showed a higher calcium concentration in the eggshell (40.46%) of the same breeders used in this study, suggesting that has an increase in yolk calcium concentration (Bonagurio et al., 2020). Moreover, these effects of CX + 25-OH-D_3_ supplementation in diets of quail breeders can in part explain the enhanced embryo development, fertile egg, and chick hatch observed in this trial.

In addition to exerting antioxidant effects, Cx has shown to boost the immune system (Karadas et al., 2016), although the mechanisms by which this occurs are still not fully elucidated. The positive effect of 25-OH-D_3_ on the immune system is well established (Yamamoto and Jørgensen, 2019); therefore, it follows that the combined supplementation of breeder diets with Cx and 25-OH-D_3_ can contribute to embryonic survival and robust immune response in 1-day-old chicks (Sunders-Blades and Korver, 2015; Johnson-Dahl et al., 2016).

Araujo et al. (2019), studying the effects of dietary supplementation Cx in combination with 25-OH-D3 on reproductive, performance, and progeny quality in broiler breeders, found that supplementation with 6 ppm Cx and 69 µg 25-OH-D3 increased egg production, total hatchability, hatchability of fertile eggs and reduce early embryo mortality. The results found by Araujo et al. (2019) are similar to results found in our experiment, demonstrating that supplementation with Cx + 25-OH-D3 may improve the reproductive performance of breeders.

Rosa et al. (2012), in studying the effects of Cx supplementation on embryonic metabolism, found that supplementation of Cobb 500 breeders with 6 ppm Cx reduced embryonic mortality, from 1.80% to 1.04% in the first 48 h and from 2.07% to 1.44% between 15 and 21 d of incubation Zhang et al. (2011). also reported that maternal supplementation with 6 ppm Cx reduced embryonic mortality from 4% to 0%.

Excessive doses of vitamin D_3_ shown to increase embryonic mortality (Soares et al., 1979; Mottaghitalab et al., 2013). Evidence has shown that embryonic mortality increased when the metabolite 1.25-OH2-D_3_ was not associated with vitamin D_3_ (Sunde et al., 1978), and that 25-OH-D_3_ was the only vitamin D_3_ metabolite to provide the same embryonic mortality indices as vitamin D_3_ when added to broiler breeder diets (Abdulrahim et al., 1979).

Overall, these results show that to improve embryonic mortality rates, diets must contain an optimal ratio of vitamin D_3_ to 25-OH-D_3_. The possible interaction between vitamin D_3_ and 25-OH-D_3_ observed in this study, corroborate with Ghasemi et al. (2019) that reported the possibility of have been an interaction between vitamin D_3_ and 1α-cholecalciferol. In the current study, the quadratic dependence of most parameters on Cx + 25-OH-D_3_ levels demonstrates the importance of defining the adequate Cx + 25-OH-D_3_ concentration.

### Chick Quality

Chick weight and length, as well as Pasgar score, could reflect the chick development during the first live weeks of chick. The quality of chick 1-day-old is essential for broiler production. Results of chick quality are described on Table 3. There were no interaction effects (*P* > 0.05) between breeder age and Cx + 25-OH-D_3_ supplementation on chick quality.

**Table 3:** Analysis of the quality of day-old chicks from European quail breeders supplemented with Cx + 25-OH-D_3_.

	Chick quality				
Items			Length, (cm)		Pasgar score
Breeder age					
32 wk	8.37		11.65^B^		9.29
38 wk	8.48		12.07^A^		9.28
Diets^1^					
Basal (50 µg D_3_)	8.26		11.59		8.98
3 Cx + 34.5 µg 25-OH-D_3_	8.63		12.01		9.38
6 Cx + 69 µg 25-OH-D_3_	8.39		11.99		9.51
9 Cx + 103.5 µg 25-OH-D_3_	8.35		11.89		9.28
12 Cx + 138 µg 25-OH-D_3_	8.49		11.82		9.29
Mean	8.42		11.86		9.29
SEM	0.080		0.049		0.420
Variation sources			*P*-value		
Age	0.456		<0.001		0.985
Linear	0.477		0.002		<0.001
Quadratic	0.544		0.003		<0.001
Interaction	0.850		0.387		0.818
Regression equations		R²	Vertex (ẏ^2^)	Cx (ppm)	25-OH-D_3_ (µg)
Chick length	Ý = 11.65 + 0.114Cx − 0.009Cx²	0.85	12.01	6.33	72.80
Pasgar score	Ý = 9.02 + 0.125Cx - 0.009Cx²	0.78	9.45	6.94	79.81

1 Basal diet (BD), containing 50 µg vitamin D_3_; 3Cx, BD supplemented with 3 ppm Cx and 34.5 µg 25-OH-D_3_; 6Cx, BD supplemented with 6 ppm Cx and 69.0 µg 25-OH-D_3_; 9Cx, BD supplemented with 9 ppm Cx and 103.5 µg 25-OH-D_3_, and 12Cx, BD supplemented with 12 ppm Cx and 138.0 µg 25-OH-D_3_ per kilogram.

2 Maximum or minimum ŷ values.

A-B Capital letters represent the differences between the mean ages that were detected (*P* < 0.05) by the least squares mean test.

Breeder age influenced length of chick (*P* < 0.01). Wherein, chick hatched from 38-wk-old breeders showed a higher length than those hatched from 32-wk-old breeders (Table 3).

It is well known that older's breeder produces heavier and higher nutritious eggs compared to younger breeders. Moreover, this effect in part can explain the higher length of chick hatched of breeders from 38 wk old.

**Figure 2:**
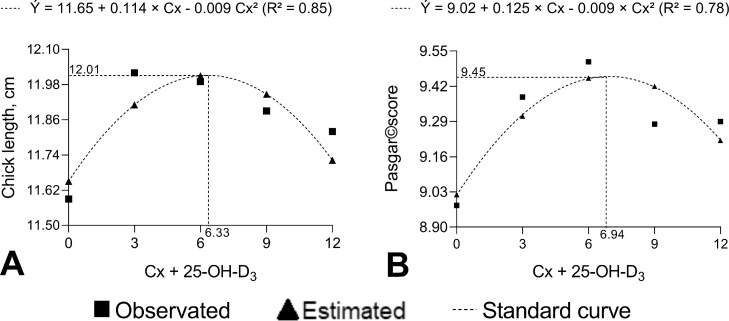
Graphics of effect of diets (Cx level is used to regression) on the chick's quality. Diets: Basal diet (BD), containing 50 µg vitamin D_3_); 3Cx, BD supplemented with 3 ppm Cx and 34.5 µg 25-OH-D_3_; 6Cx, BD supplemented with 6 ppm Cx and 69.0 µg 25-OH-D_3_; 9Cx, BD supplemented with 9 ppm Cx and 103.5 µg 25-OH-D_3_, and 12Cx, BD supplemented with 12 ppm Cx and 138.0 µg 25-OH-D_3_ per kilogram.

Cx + 25-OH-D_3_ level had a quadratic positive effect on 1-day-old chick length (*P* < 0.01), and Pasgar score (*P* < 0.01). The highest length of chick (12.15 cm) and Pasgar score (9.45), could achieve with 6.33 ppm Cx + 72.80 µg 25-OH-D_3_, and 6.94 ppm Cx + 79.81 µg 25-OH-D_3_, respectively (Table 3 and Figure 2A and B).

The increase in chick length and Pasgar score with Cx + 25-OH-D_3_ levels suggests that supplementation contributed to embryonic development and chick quality. In part, due to the effect of Cx in scavenging free radicals in several tissues, promoting a reduce in oxidative stress on embryo metabolism, and in peroxidation of nutrients following at period of egg incubation. Furthermore, the effect of Cx associated with 25-OH-D_3_ on enhancing the immune system of the embryo is another important role (Esatbeyoglu and Rimbach, 2016), which needs emphasizing in the newest research. It could partially explain the increased embryo development, reflected in augmenting in length, and Pasgar score of chick 1-day-old.

In addition, 25-OH-D_3_ could improve calcium structure eggshells. It provides a reduction in damage caused by pathogens as embryo development following. Therefore, Cx associated with 25-OH-D_3_ can enhance the quality of chick 1-day-old, a fact that benefits broiler growth.

### Analysis of Sperm–Egg Interaction

**Figure 3:**
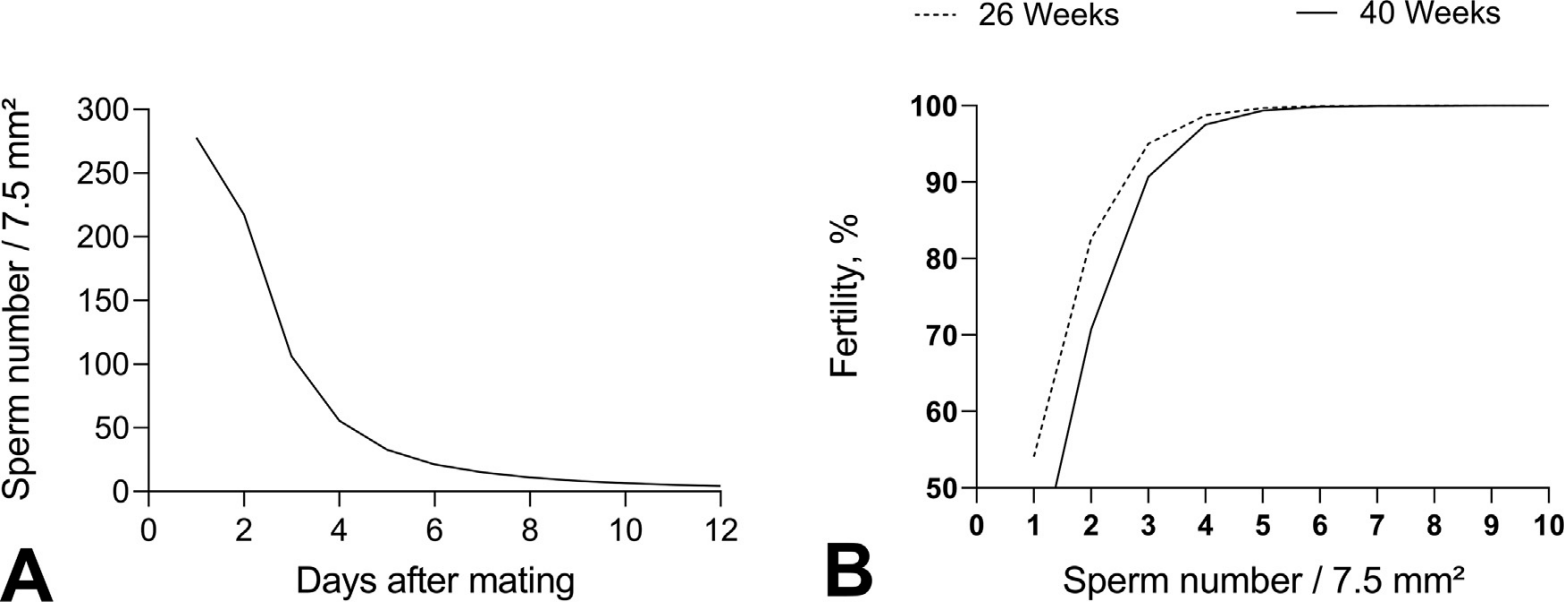
Analysis of the total number of spermatozoids fixed on the perivitelline membrane as a function of the days of egg collection after mating of European quail breeders (A). Probability of fertility in European quail breeders, as a function of the total number of spermatozoa attached to the perivitelline membrane counted in an area of 7.5mm^2^ overlying the germinal disc (B). Sperm number (β) = $e^{\beta }$; (β) = 0.0064 − 0.0047 Day + 0.0019 Day² (*P* < 0.001). Probability of fertility (β) = [1 −$e^{\beta }$/$\left(1+e^{\beta }\right)$)] × 100. Values of (β): 26 weeks (β) = 1.228 − 1.393 Sptz (*P* < 0.001). 40 weeks (β) = 1.903 − 1.393 Sptz (*P* <0.001).

The number of sperms trapped in the perivitelline membrane following the days after mating, and the probability of fertile analyzed in function of the sperm number indicates the number of sperm need and days that sperm can fertilize the oocyte. The probability of fertility was analyzed in eggs from breeders after a period of 24 h in egg collected during consecutive days. Results from those eggs can be analyzed in different form (Figure 3).

When we estimate the number of sperm obtained in function of day of egg after the mating day, the results obtained in all egg independent of treatment to European quail breeders was a quadratic (*P* < 0.01) behavior with a strong decrescent curve (see Figure 3A). In the first egg obtained in day zero after the mating period (24 h) the total sperm estimation was above 290 spermatozoids. After 6 consecutive days (eggs) these number reduced below 30 spermatozoids and after 9 days reduced to less than 02 sperm cells (Figure 3A).

Probability of an egg be fertile was estimated in function of day after mating period (24 h) and also by age of breeders (26 and 40 wk) (see Figure 3B). The probability was significatively different (*P* < 0.01) between ages and youngest breeders (26 wk) had a higher probability of fertile eggs when compared with results of older breeders (40 wk). Results indicated that when 3 sperm/mm² are over the germinal disc region there is a 97% of probability of the egg being fertile. After 5 sperm cells there is 100% of probability of fertility (see Figure 3B).

Our findings agree with Santos et al. (2015) who reported that older male breeder ages (39 wk vs. 20 wk) negatively affect hatchability of fertile eggs, egg production, egg weight, and late embryonic mortality but not fertility. The authors also evaluated egg–sperm interactions and found that female breeder age influenced the number of sperm cells attached to the perivitelline membrane 8 d after mating, suggesting that female age directly interferes with fertility maintenance in European quail (Santos et al., 2013).

In addition to reduced sperm cell count, older age is associated with decreased semen quality (Bramwell et al., 1996), ejaculate volume (Zhang et al., 1999), sperm cell concentration (Sexton et al., 1989), and ability of sperm cells to penetrate the perivitelline membrane (Bramwell et al., 1996). A decrease in the antioxidant capacity of females as a function of age may expose stored sperm cells to oxidation (Surai et al., 1998).

### Analysis of Sperm–Egg Interaction: Probability of Fertility in Function of Day After Mating

**Figure 4:**
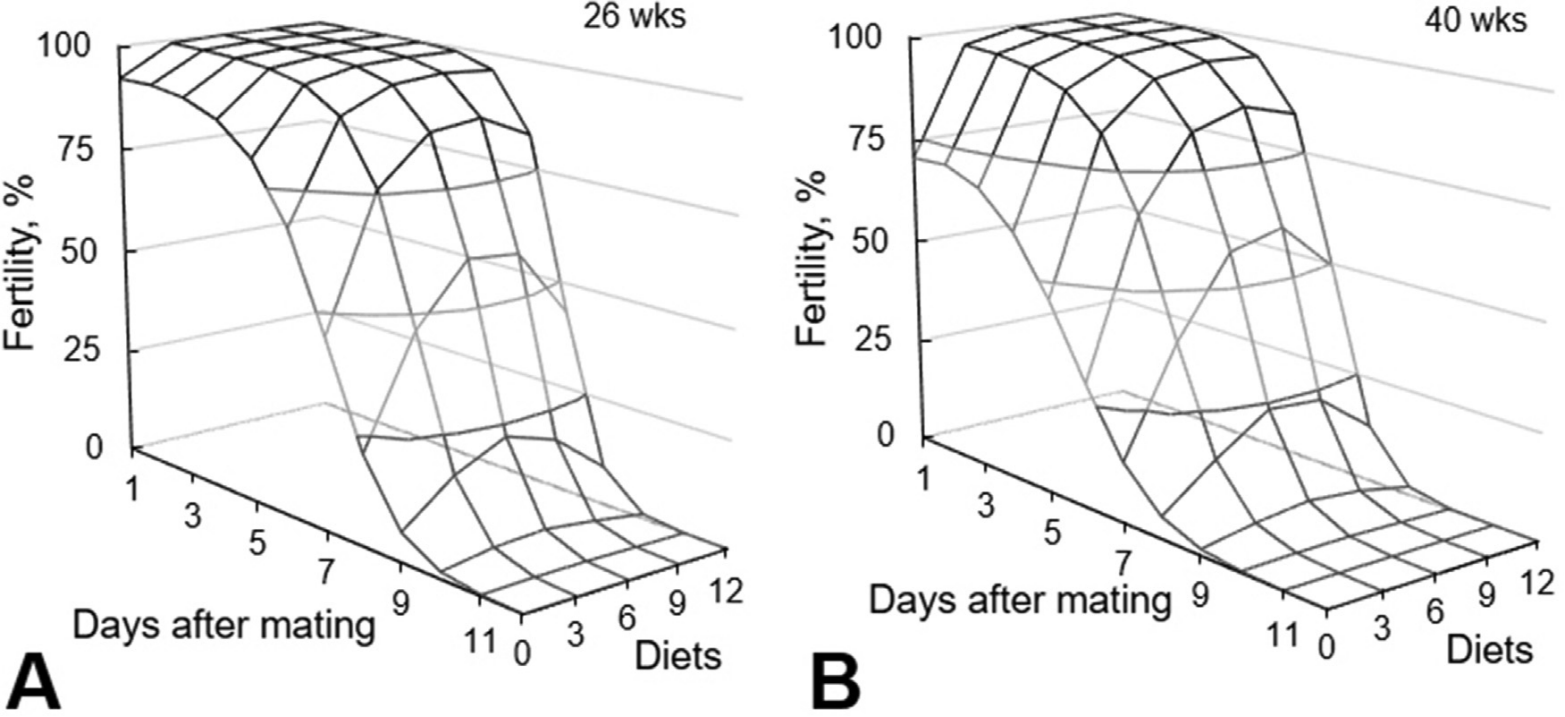
Probability of fertility estimated in consecutive eggs after mating of breeders at 26 and 40 weeks of age analyzed by morphology of the germinal disc. Probability of fertility = *e^β^*/(1 + *e^β^*). Values of (β): 26 wk (β) = −2.263 − 0.989 Diets + 0.034 Diets² − 0.308 Days + 0.094 Days² + 0.062 × Diets × Days *P* < 0.001). 40 wk (β) = −0.668 − 0.989 Diets + 0.034 Diets² − 0.308 Days + 0.094 Days² + 0.062 Diets × Days (*P* < 0.001). Diets: Basal diet (BD), containing 50 µg vitamin D_3_); 3Cx, BD supplemented with 3 ppm Cx and 34.5 µg 25-OH-D_3_; 6Cx, BD supplemented with 6 ppm Cx and 69.0 µg 25-OH-D_3_; 9Cx, BD supplemented with 9 ppm Cx and 103.5 µg 25-OH-D3, and 12Cx, BD supplemented with 12 ppm Cx and 138.0 µg 25-OH-D_3_ per kilogram.

Probability of fertility in the function of days after mating indicates the capacity of female stored and maintain viable the sperms after mating, to fertilize the oocyte. The probability of fertility on the consecutive egg where also analyzed in function of day after mating and of treatments. The probability fertility of egg was determined by the morphology of the germinal disc. Age influenced (*P* < 0.01) probability fertility of egg and a distinct behavior were observed between curves obtained to effect of treatment and diets interaction in results from breeders with 26 (Figure 4A) or 40 wk (Figure 4B). No interaction effects between Cx associated with 25-OH-D_3_ and breeder age were observed.

The interaction between Cx associated with 25-OH-D_3_ × days after mating had a negative quadratic effect on the fertility (*P* < 0.01) of eggs in both ages (Figure 4A and B). The highest fertility (>99.99%) was estimated to be achieved with 8.69 ppm Cx + 99.9 µg 25-OH-D_3_ on d 2 after copulation. A higher level of Cx + 25-OH-D_3_ than estimated could reduce fertility on d 5 after mating.

The egg fertility of supplemented breeders aged 26 wk was 80 to 100% u*P* to 7 d after mating, except in breeders supplemented with 12 ppm Cx + 138 µg 25-OH-D_3_, which showed egg fertility of 60 to 80% up to 5 d after mating. Egg fertility decreased to 0 to 20% on day 9 after mating (Figure 4A). According to the number of sperm cells (Figure 3A), eggs produced after this period were no longer fertile. The egg fertility of breeders fed the basal diet was 60 to 80% on d 4 after mating (Figure 4A).

For breeders aged 40 wk, supplementation resulted in an egg fertility of 80 to 100% up to 5 d after mating, decreasing to 0 to 20% only on d 8 after mating (Figure 4B). Eggs were no longer fertile on d 8 after mating, according to the sperm cell count (Figure 3A). The egg fertility of control group breeders was 60 to 80% on d 1 after mating and 40 to 60% on d 4 (Figure 4B). These results are in agreement with the findings of Santos et al. (2013), who showed that egg fertility remains above 20% up to 9 d after mating.

In this study, the probability of egg fertility was not affected by age, probably as a result of the antioxidant effects of Cx. The Cx may have reduced sperm peroxidation in older birds. This hypothesis was confirmed by the similar fertility rates of 40- and 26-wk-old breeder eggs up to 5 d after mating (Figure 4).

The high egg fertility observed in this study was likely associated with the positive effect of Cx + 25-OH-D_3_ levels on the number of sperm cells in the germinal disc. Our previously published study has demonstrated that dietary breeder supplemented with 5.81 ppm of Cx + 87.88 µg of 25-OH-D_3_ showed a higher (*P* = 0,004) expression of SOD1 (0.249), and a response of linearly increase the expression of GPx-7 (*P* = 0.007) in the oviduct trait of female quail relied the added higher levels of Cx + 25-OH-D_3_ in diets (Bonagurio et al., 2020). It could be partially explaining the higher probability of egg fertility founded in the present trial.

Several trials have indicated that SOD and GPx together with Cx create a network by preventing and scavenging free radicals from several tissues, such as the phospholipid membrane, as well as from the female reproductive tract, thereby decreasing PUFAs peroxidation (Surai, 2016; Elia et al., 2019). Moreover, these effects of Cx on defense on antioxidant system could increase sperm cell viability and longevity.

### Analysis of Sperm–Egg Interaction: Total Sperm Cells Trapped on the Perivitelline Membrane

The measurement of total sperm cells trapped on the perivitelline membrane can indicate if the experimental diets promote effects on the survival of sperms in the oviduct trait, such as the probability of fertility. Total sperm cells trapped on the perivitelline membrane were counted over the germinal disc area in the same eggs where fertility was determined.

**Figure 5:**
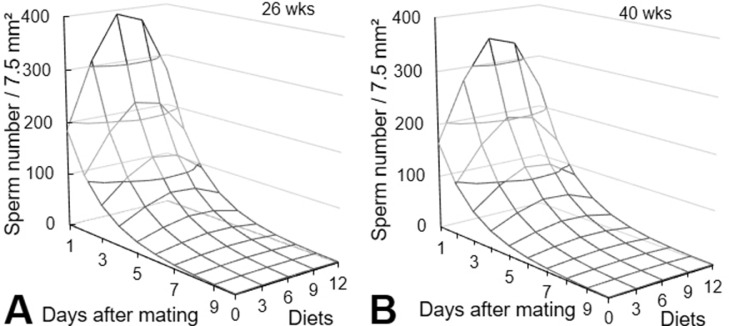
Estimated sperm number fixed on the perivitelline membrane depending on the age of the breeders (26 and 40 wk), the days on which the eggs were collected after mating of breeders and the levels of Cx + 25-OH-D_3_ (diets). Sperm quantity was measured in an area of 7.5 mm² overlaying the germinal disc of perivitelline membrane. Total sperm number = $e^{\beta }$; Estimated β values: 26 wk (β) = 5.751 + 0.2173 Diets − 0.0155 Diets² − 0.5216 Days − 0.0034 Days² (*P* < 0.001). 40 wk (β) = 5.631 + 0.2173 Diets − 0.0155 Diets² − 0.5216 Days − 0.0034 Days² (*P* < 0.001). Diets: Basal diet (BD) with 50 µg vitamin D_3_; 3Cx, BD supplemented with 3 ppm Cx and 34.5 µg 25-OH-D_3_; 6Cx, BD supplemented with 6 ppm Cx and 69.0 µg 25-OH-D_3_; 9Cx, BD supplemented with 9 ppm Cx and 103.5 µg 25-OH-D_3_, and 12Cx, BD supplemented with 12 ppm Cx and 138.0 µg 25-OH-D_3_ per kilogram.

There was interaction (*P* < 0.05) of breeder age, days after mating, and treatments on the number of sperm cells attached to the perivitelline membrane (Figure 5). Sperm cell number decreased linearly with days after mating (*P* < 0.01) and increased quadratically with Cx + 25-OH-D_3_ levels (*P* < 0.01). The highest sperm cell number was estimated to be reached with 6.54 ppm Cx + 75.2 µg 25-OH-D_3_ (Figure 5).

The results indicate that the eggs from breeders aged 26 wk had a higher sperm cell count than those from breeders aged 40 wk (Figure 5A). However, we highlight that the sperm cell number in eggs from older breeders supplemented with 3 ppm Cx + 34.5 µg 25-OH-D_3_ and 6 ppm Cx + 69 µg 25-OH-D_3_ was similar to that of eggs from 26-wk-old breeders (Figure 5B). It is noticeable that these results clearly showed a strong effect on oldest breeders, this could be by an increment on sperm survival inside sperm tubules in vagina mucosa.

Cx + 25-OH-D_3_ supplementation, therefore, helped maintain egg fertility in 40-wk-old breeders, in agreement with reports showing that protection of lipid membranes against peroxidation increases in vivo and in vitro sperm cell survival (Surai et al., 1998; Surai, 2002; Bréque et al., 2006).

Our results corroborate those obtained with Cobb 500 breeders: the sperm cells of broilers supplemented with 6 ppm Cx showed increased longevity in the female reproductive tract (Rosa et al., 2012) Bréque et al. (2006). pointed out that the glands of the uterovaginal junction protect sperm cells against lipid oxidation by secreting antioxidants, such as vitamins E and C, carotenoids, superoxide dismutase, glutathione peroxidase, and catalase. Cx was essential for the protection of the phospholipid layers of sperm cells that were not affected by age-related oxidation, increasing fertility.

It has been established that the number of sperm is correlated with the probability of fertility, and also is an indication of sperm quality (Findeklee et al., 2020). Therefore, the increased in the number of sperm cells reached the infundibulum of the uterine tube of breeder supplemented with Cx + 25-OH-D_3_ level, may also have increased ejaculate volume and storage conditions in the sperm storage tubules.

Vitamin D_3_ and 25-OH-D_3_ are precursors of the active hormone calcitriol (1,25-OH_2_-D_3_), which has anti-inflammatory and immune-stimulating activities (Jeon and Shin, 2018). The hormone modulates T-cell function and plays a role in a variety of biological processes, including those related to reproduction. Its deficiency is associated with male infertility and embryonic malformation (Hewison, 2012; Rudick et al., 2014). In birds, there is little information on the role of vitamin D in fertility; however, in rats, it was shown that vitamin D deficiency or absence of receptors leads to uterine malformation, resulting in infertility (Luk et al., 2012).

## CONSIDERATIONS

We observed quadratic effects on reproductive performance and chick quality in function of Cx + 25-OH-D3 supplementation. These results showed that the supplementation of Cx + 25-OH-D3 in optimal levels in quail breeder's diets is essential to maximize reproductive performance and chick quality. On the other hand, it is necessary to emphasize that after the optimal levels occurred a reduction in these parameters.

In part, these negative effects may be explained in functions of pro-oxidants activity of Cx. The literature showed that the Cx and other β-carotene have oxidant and pro-oxidant actions according to the levels used (Surai et al., 2001; Black et al., 2020; Shin et al., 2020) Surai et al. (2001)., mentioned that the antioxidant activity of carotenoids may shift into pro-oxidant activity depending on the redox potential of the carotenoid molecules as well as on the biologic environment in which they act. According to Black et al. (2020). In dietary β-carotene supplement studies, damaging pro-oxidant reactivity can also arise. Reasons for this switch are likely due to the properties of the carotenoid radicals themselves. Understanding singlet oxygen reactions and the anti-/pro-oxidant roles of carotenoids are of importance to photosynthesis, vision, and cancer.

The literature about the Cx + 25-OH-D3 supplementation in quail breeder's diet is scarce, it is necessary to develop new researches with Cx + 25-OH-D_3_ supplementation in quail breeder's diets to understand these negative effects on reproductive performance and chick quality.

## CONCLUSION

Overall, the results showed that supplementation of European quail breeder diets with 5 to 6 ppm Cx and 57.50 to 69 µg 25-OH-D_3_ enhances hatchability of total and fertile eggs, fertility, chick quality sperm and cell longevity in sperm storage tubules, especially in older quail's breeders and reduces embryonic mortality.
